# Supplementary material for: Beyond correlation: optimal transport metrics for characterizing representational stability and remapping in neurons encoding spatial memory
Source: Front Cell Neurosci. 2024 Jan 11;17:1273283. doi: 10.3389/fncel.2023.1273283 (PMC10831886; doi:10.3389/fncel.2023.1273283)
Supplement: Supplementary file 1 [file Data_Sheet_1.pdf]

Supplementary Material

1 SUPPLEMENTARY DATA

Additional examples of synthesized field data pre-interpolation. Data are generated using basic place field manipulations to vary location, size and shape. Examples of corner/directional fields, field scaling and field tracking using EMD and centroid distances for the 4 cardinal directions and angles.

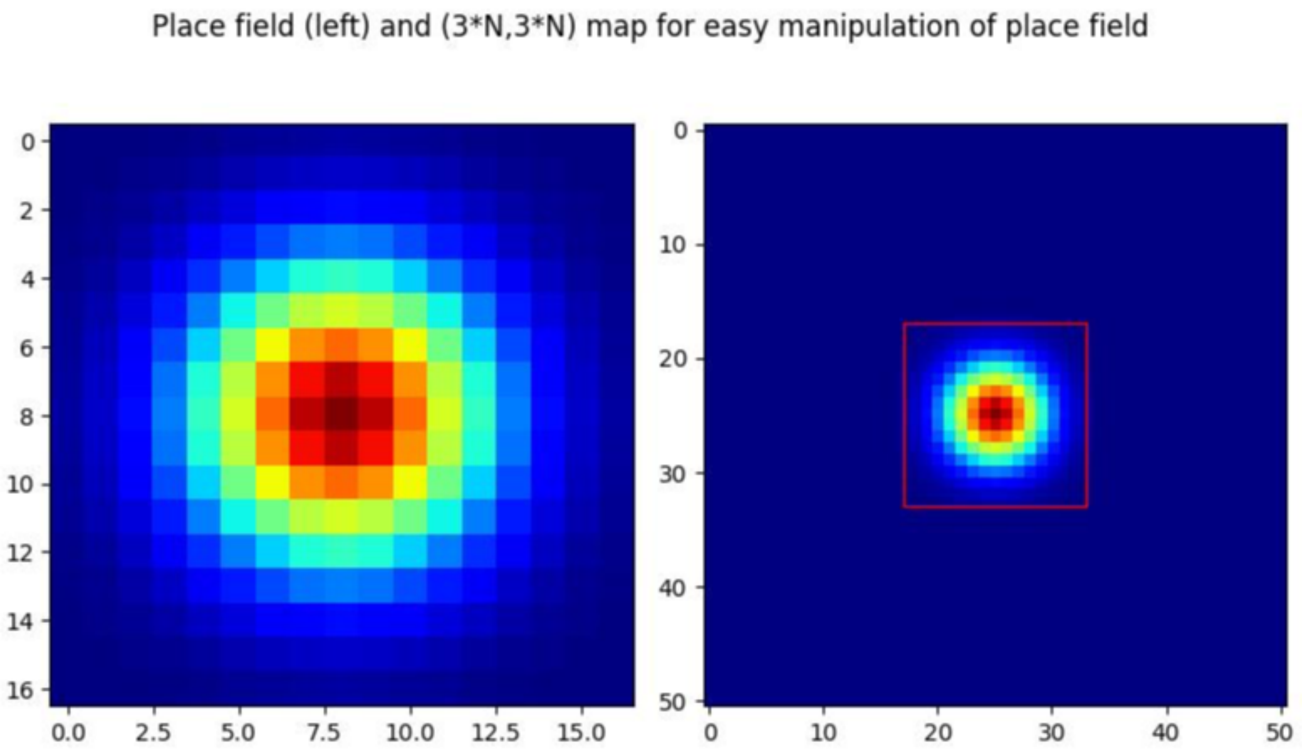

Figure S1. Synthetic gaussian field. Example of a (17,17) gaussian field with $\sigma = 3$ without interpolation to (257,257) (left). A wider (3*17,3*17) map is shown which allows for shifting of the gaussian field by its centroid (right). The red square denotes all possible locations a centroid can move to.

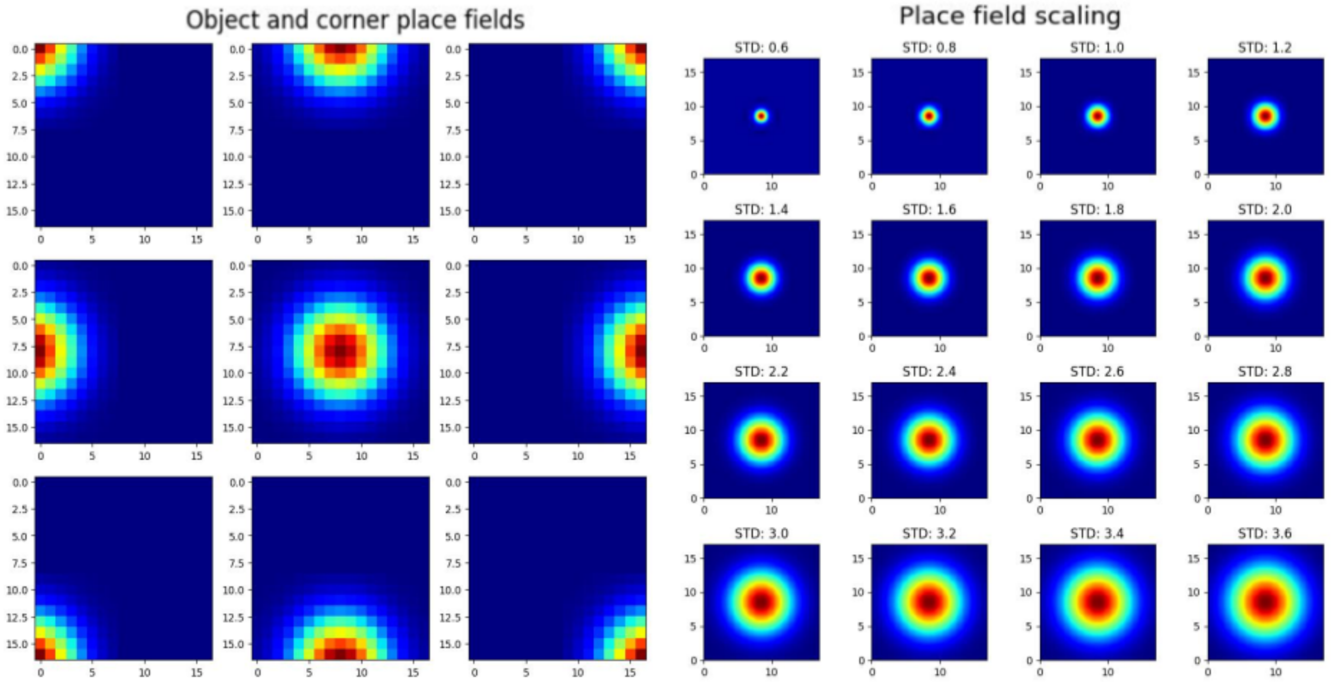

Figure S2. Non-linear field transformations. Corner/boundary fields are shown where consecutive 90 degree transformations create 4 rotated positions for each field in the square arena (left). Example of scaling by varying field σ from 0.6 to 3.6 (right).

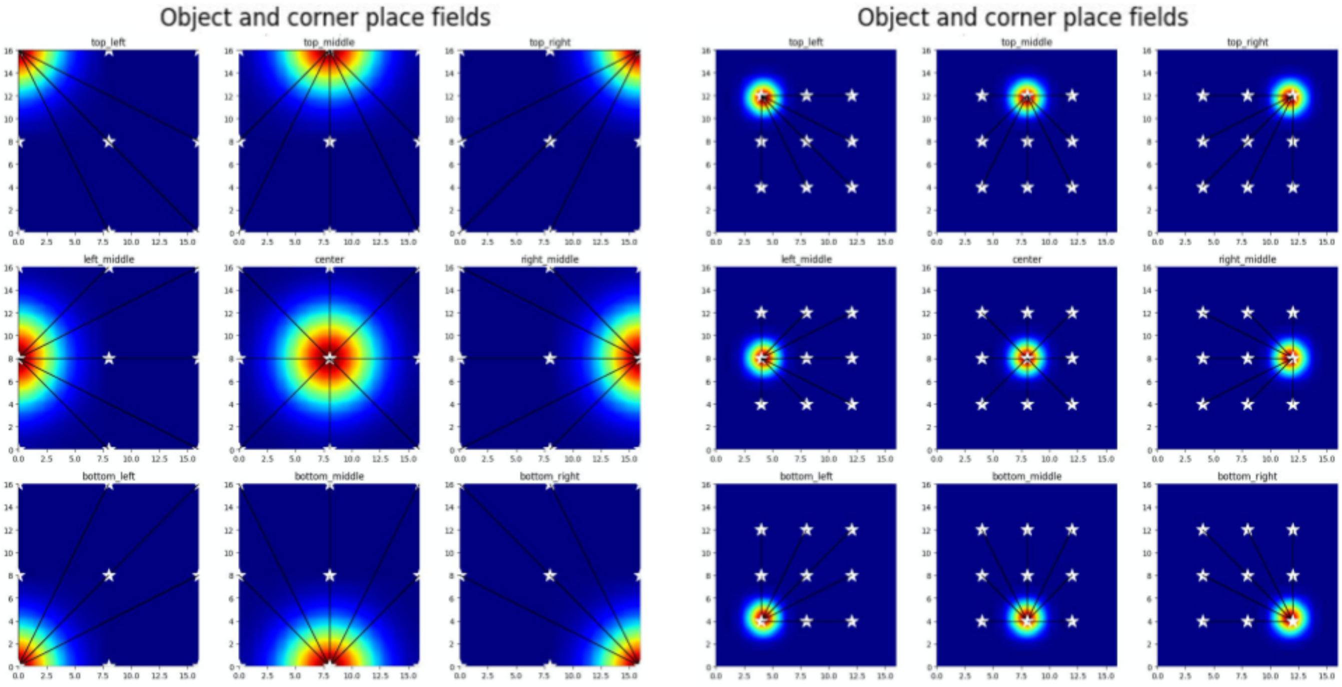

Figure S3. Object/Trace transformations. Example of fields to be used for rotational remapping and object/trace mechanisms. Plots show fields at the 4 corners, the 4 cardinal directions (N,S,W,E) and the center. Object locations are denoted by white stars and connected to the field centroid with a black line (centroid distance).

2 SUPPLEMENTARY TABLES AND FIGURES

2.1 Figures

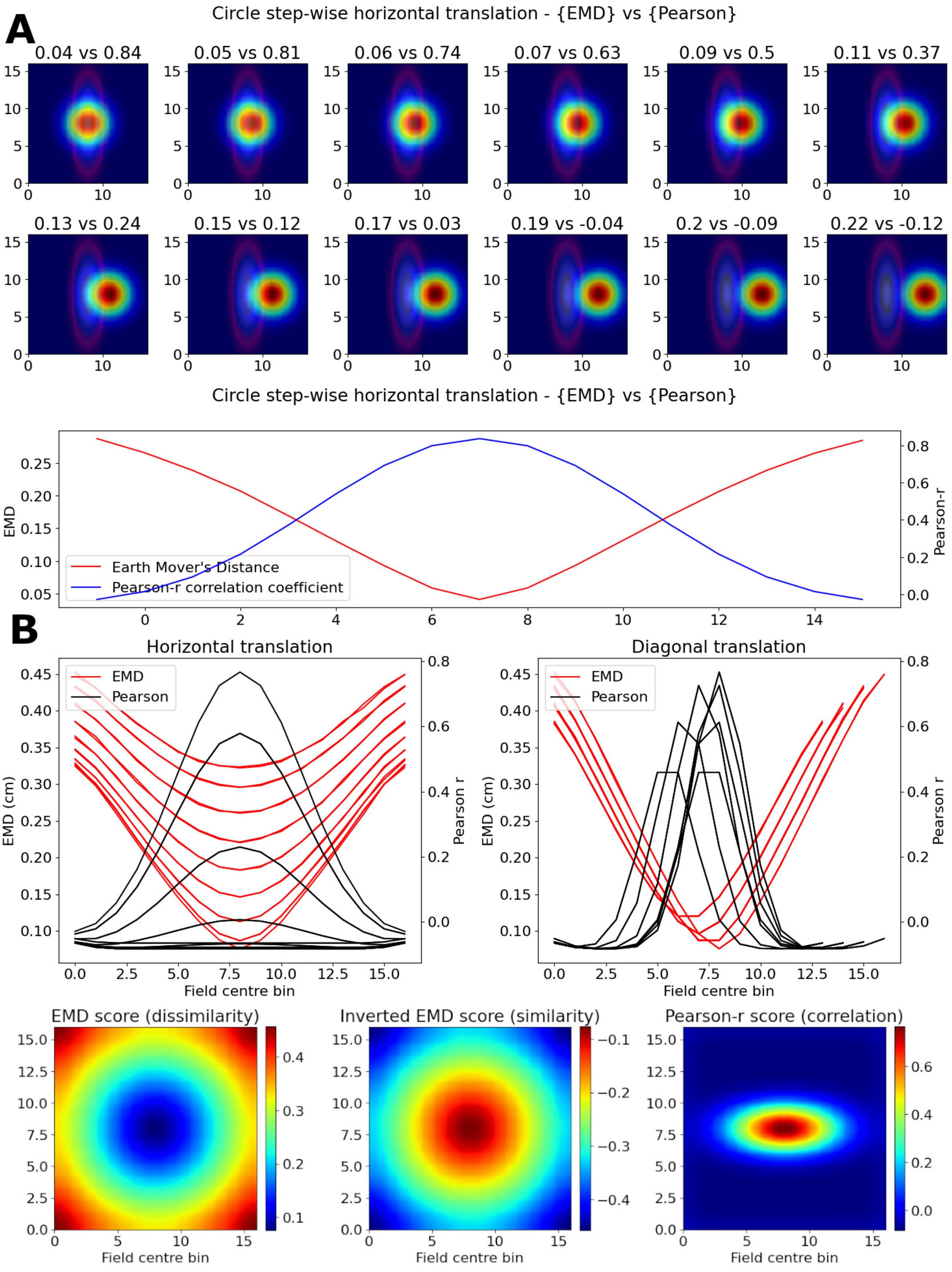
A
Circle step-wise horizontal translation - {EMD} vs {Pearson}
0.04 vs 0.84
0.05 vs 0.81
0.06 vs 0.74
0.07 vs 0.63
0.09 vs 0.5
0.11 vs 0.37
0.13 vs 0.24
0.15 vs 0.12
0.17 vs 0.03
0.19 vs -0.04
0.2 vs -0.09
0.22 vs -0.12
Circle step-wise horizontal translation - {EMD} vs {Pearson}
EMD
Pearson-r
Earth Mover's Distance
Pearson-r correlation coefficient
B
Horizontal translation
Diagonal translation
EMD
Pearson
EMD (cm)
Pearson r
Field centre bin
EMD score (dissimilarity)
Inverted EMD score (similarity)
Pearson-r score (correlation)
Field centre bin

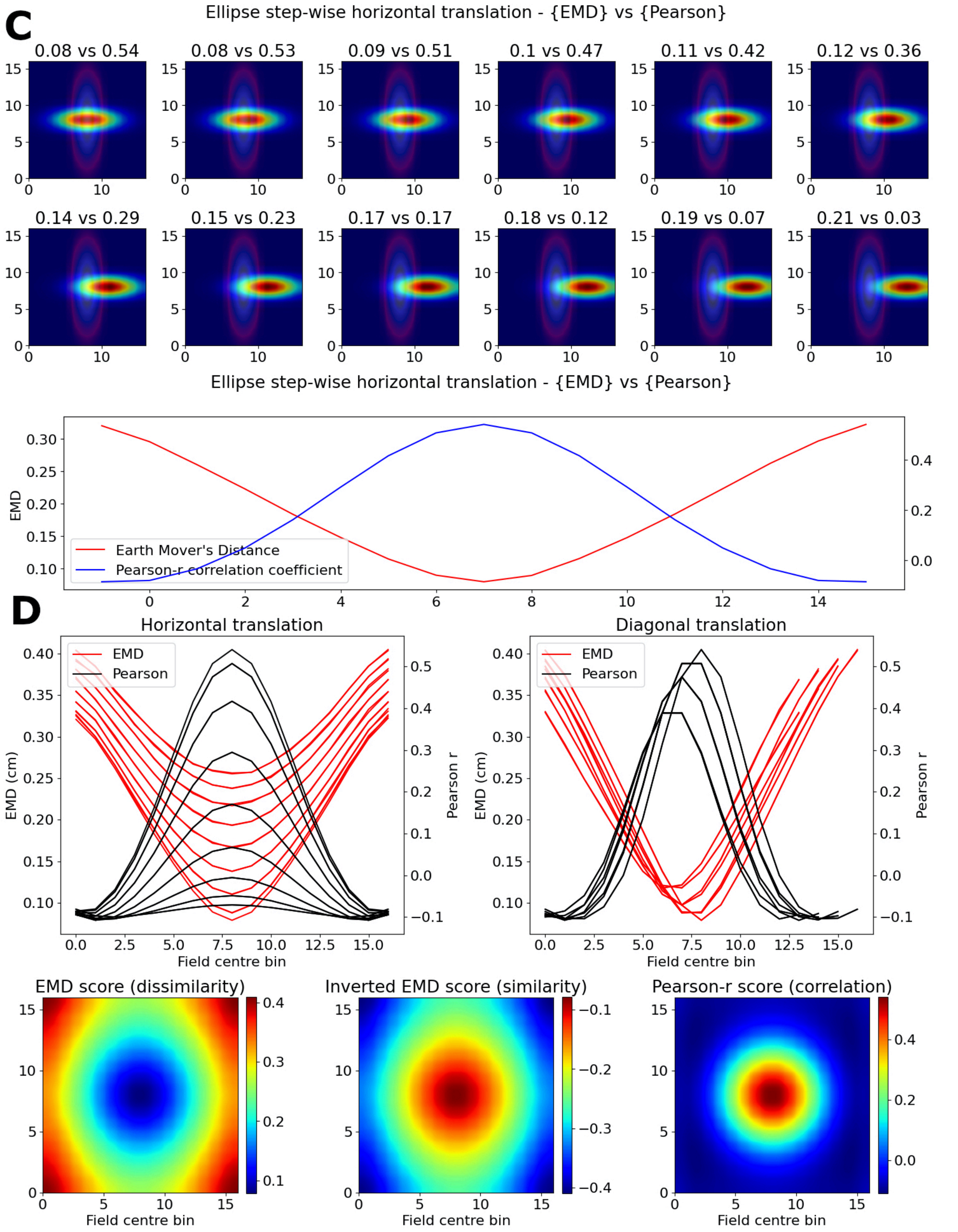

Figure S4. Non-identical place field translation. Stepwise horizontal linear translation of non-identical, overlapping place fields ($N = 17$, $\sigma = 3$) moving from the center to the right (**A, C**). EMD score is shown on the left while Pearson's r is shown on the right. 12 steps are shown and scores are rounded for display (top panel). Scores from remapping tested at all possible centroids in a single row on the rate map (bottom panel). EMD and Pearson's r scores tested at all possible centroids in the rate map (N*N) (**B, D**). Horizontal and diagonal translations across the rate map are shown for all rows ($N = 17$) (top panel). Heatmap showing the gradient of EMD scores both raw and inverted to match Pearson's r color scheme (bottom panel).

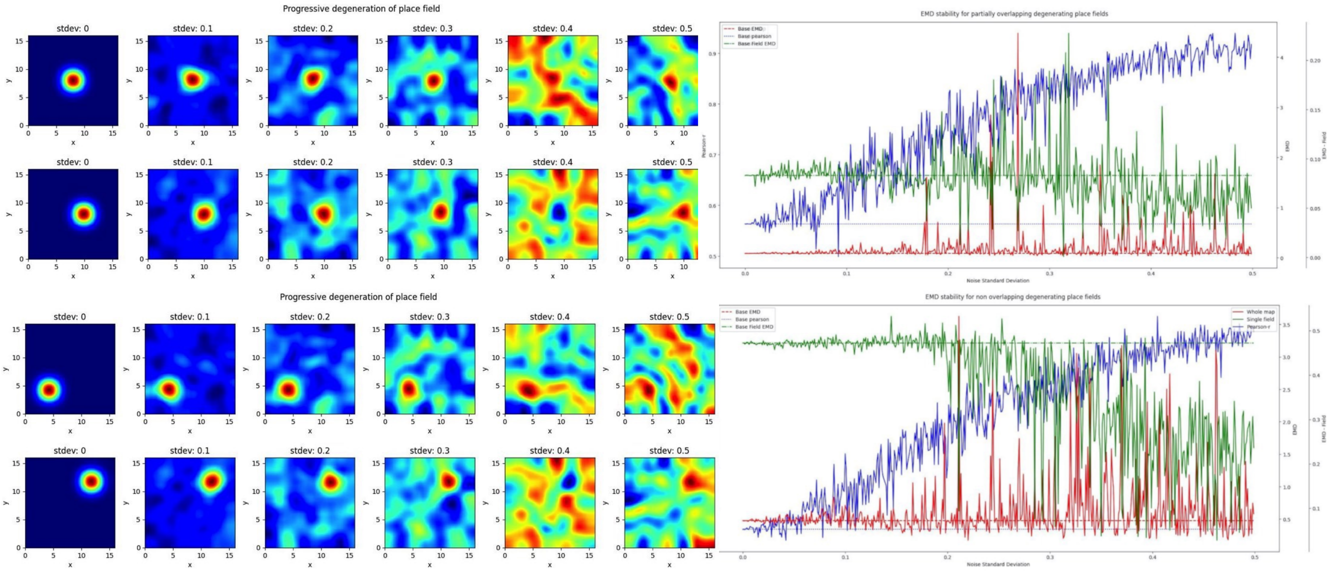

Figure S5. Repetition of Figure 3 but for normalization and smoothing post-added noise. The same noise distribution that was added to the unnormalized case is added at each step. Examples are shown for overlapping (top panel) and non-overlapping fields (bottom panel).

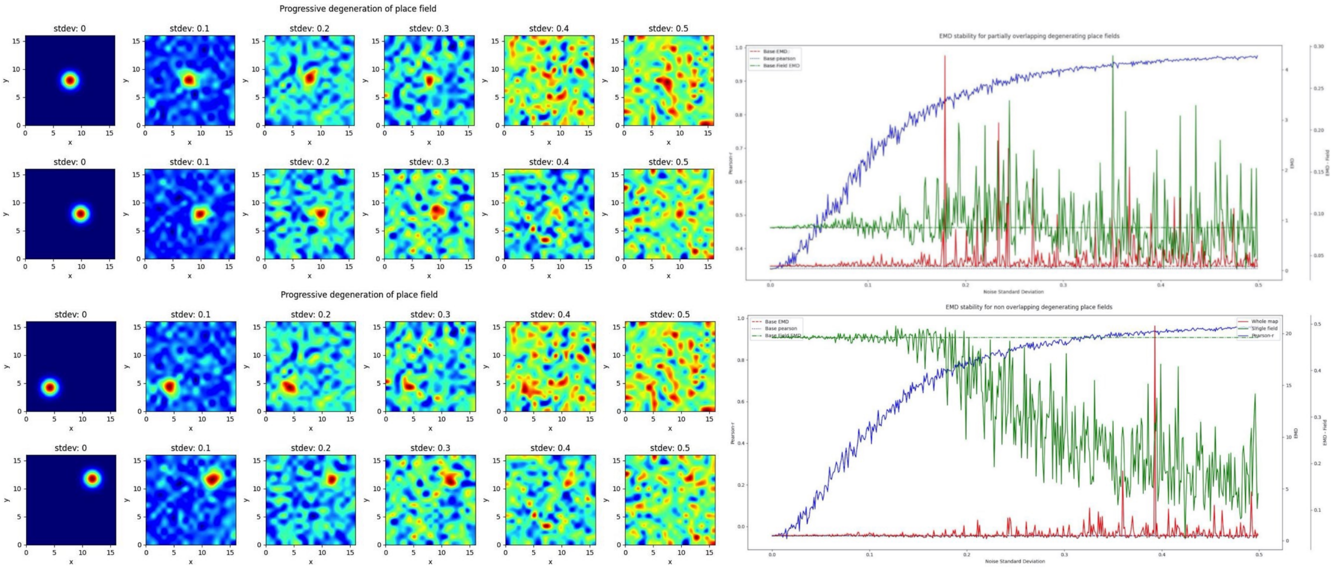

Figure S6. Repetition of Figure 3 but for normalization only post-added noise. The same noise distribution that was added to the unnormalized case is added at each step. Examples are shown for overlapping (top panel) and non-overlapping fields (bottom panel).

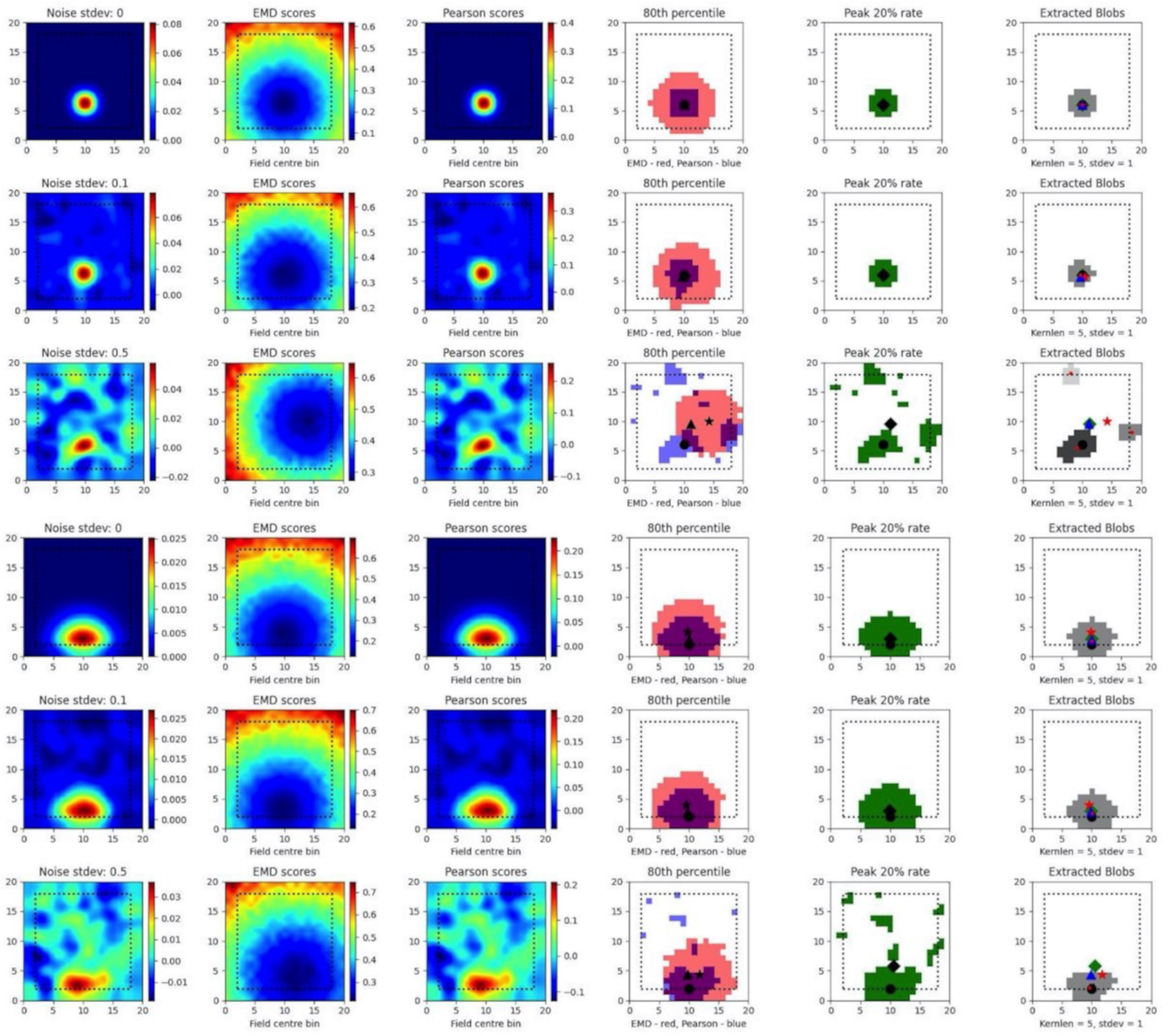

Figure S7. Single field localization. Additional field localization plots from Figure 4). Here, localization plots are shown across three different noise levels (rows: no noise 0, low noise 0.1 and high noise 0.5)

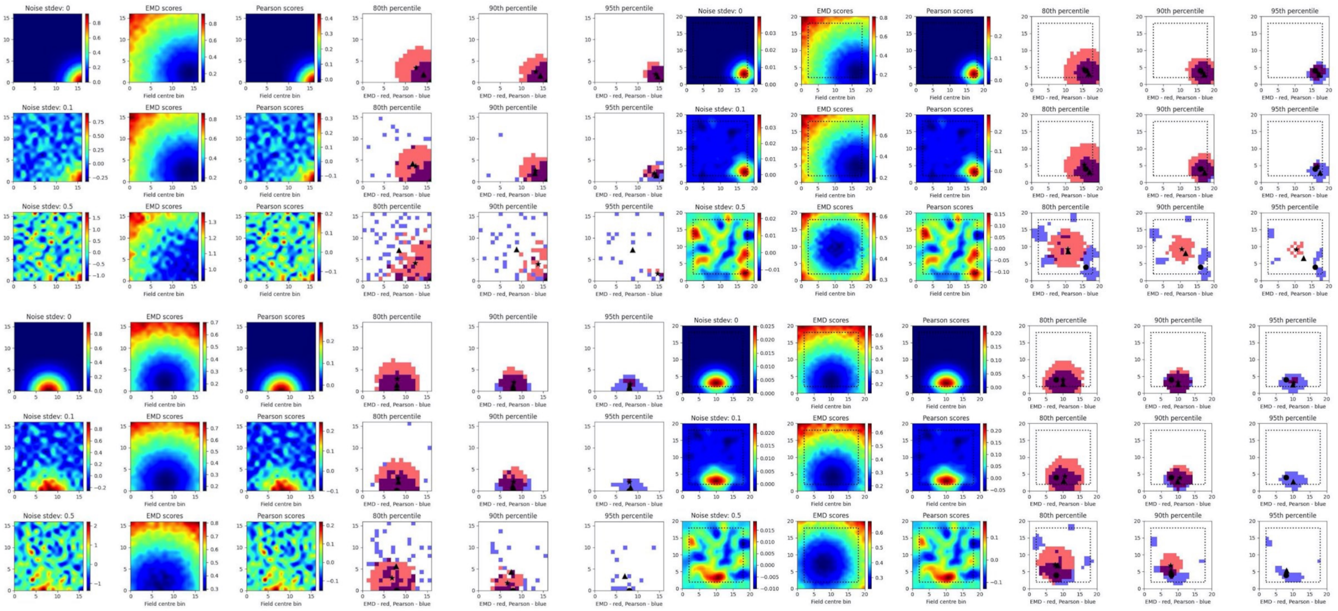

Figure S8. Localization with padding and smoothing. These plots come in pairs where the first 3 rows (no noise 0, low noise 0.1 and high noise 0.5) are the unnormalized, unsmoothed and unpadded version of Fig4 while the next 3 rows show the recovery of scores (in some cases) post padding, normalizing and smoothing. The last 3 columns are for the specificity of scores and show the 80th, 90th and 95th percentile of EMD and Pearson's r scores.
